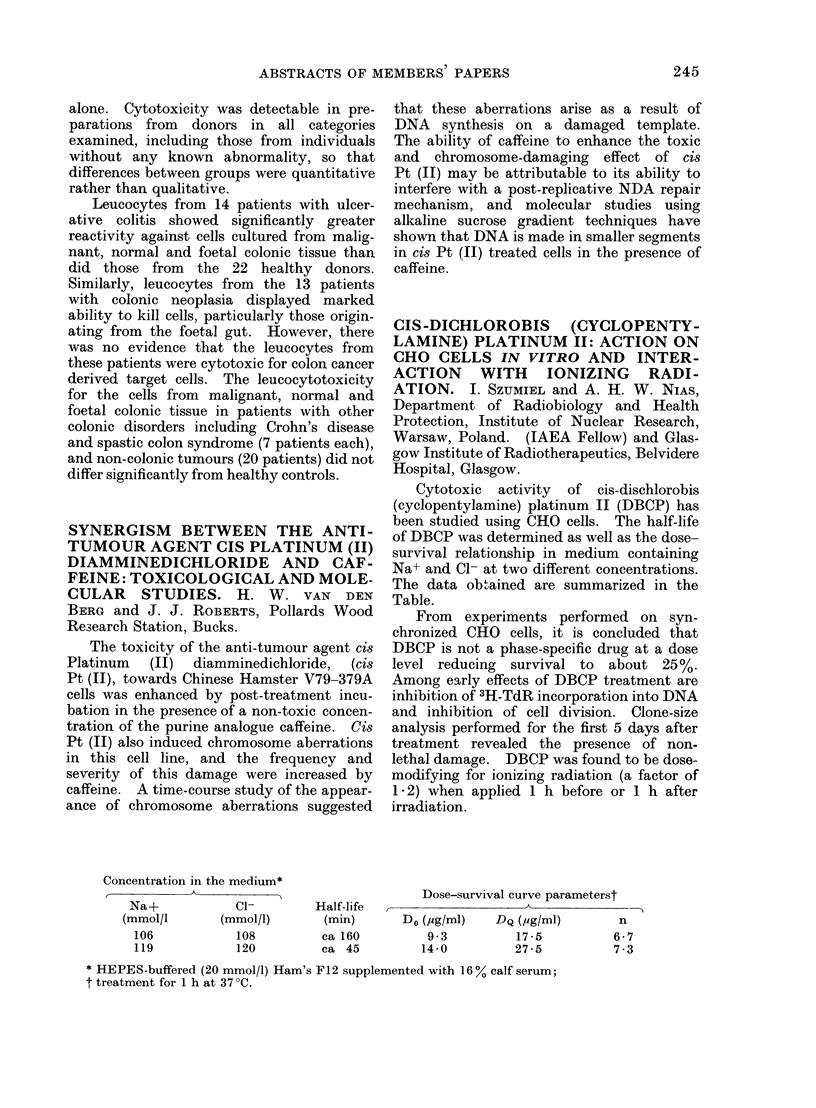# Proceedings: Synergism between the anti-tumour agent cis platinum (II) diamminedichloride and caffeine: toxicological and molecular studies.

**DOI:** 10.1038/bjc.1975.171

**Published:** 1975-08

**Authors:** H. W. van den Berg, J. J. Roberts


**SYNERGISM BETWEEN THE ANTI-TUMOUR AGENT CIS PLATINUM (II) DIAMMINEDICHLORIDE AND CAFFEINE: TOXICOLOGICAL AND MOLECULAR STUDIES.** H. W. van den Berg and J. J. Roberts, Pollards Wood Research Station, Bucks.

The toxicity of the anti-tumour agent *cis* Platinum (II) diamminedichloride, (*cis* Pt (II), towards Chinese Hamster V79–379A cells was enhanced by post-treatment incubation in the presence of a non-toxic concentration of the purine analogue caffeine. *Cis* Pt (II) also induced chromosome aberrations in this cell line, and the frequency and severity of this damage were increased by caffeine. A time-course study of the appearance of chromosome aberrations suggested that these aberrations arise as a result of DNA synthesis on a damaged template. The ability of caffeine to enhance the toxic and chromosome-damaging effect of *cis* Pt (II) may be attributable to its ability to interfere with a post-replicative NDA repair mechanism, and molecular studies using alkaline sucrose gradient techniques have shown that DNA is made in smaller segments in *cis* Pt (II) treated cells in the presence of caffeine.